# A disease-related gene mining method based on weakly supervised learning model

**DOI:** 10.1186/s12859-019-3078-9

**Published:** 2019-12-02

**Authors:** Han Zhang, Xueting Huo, Xia Guo, Xin Su, Xiongwen Quan, Chen Jin

**Affiliations:** 1 College of Artificial Intelligence, Nankai University, Tongyan Road, Tianjin, 300350 People’s Republic of China; 2 College of Computer Science, Nankai University, Tongyan Road, Tianjin, 300350 China

**Keywords:** Weakly supervised learning model, Differentially expressed genes, Disease-related genes, Transductive support vector machine, The difference kernel function

## Abstract

**Background:**

Predicting disease-related genes is helpful for understanding the disease pathology and the molecular mechanisms during the disease progression. However, traditional methods are not suitable for screening genes related to the disease development, because there are some samples with weak label information in the disease dataset and a small number of genes are known disease-related genes.

**Results:**

We designed a disease-related gene mining method based on the weakly supervised learning model in this paper. The method is separated into two steps. Firstly, the differentially expressed genes are screened based on the weakly supervised learning model. In the model, the strong and weak label information at different stages of the disease progression is fully utilized. The obtained differentially expressed gene set is stable and complete after the algorithm converges. Then, we screen disease-related genes in the obtained differentially expressed gene set using transductive support vector machine based on the difference kernel function. The difference kernel function can map the input space of the original Huntington’s disease gene expression dataset to the difference space. The relation between the two genes can be evaluated more accurately in the difference space and the known disease-related gene information can be used effectively.

**Conclusions:**

The experimental results show that the disease-related gene mining method based on the weakly supervised learning model can effectively improve the precision of the disease-related gene prediction compared with other excellent methods.

## Background

Neurological diseases put a serious threat to the health of human being. To explore the pathology of neurological diseases, it is promising to work on the identification of functional genes or disease-related metabolic pathways based on gene expression dataset [1, 2].

In the past, most of the researchers tended to use those basic statistical methods [3–6] to screen differentially expressed genes. The t-test method [7] and significance analysis of microarrays (SAM) [8] are usual methods. The t-test method compares the difference between disease samples and normal samples on gene expression to screen differentially expressed genes. However, the estimation of total variance is not accurate due to the small sample size. SAM controls the false discovery rate (FDR) to correct the false positive rate in multiple hypothesis testing, while the outcome is not satisfying. Nowadays, it is more often to use feature selection algorithms [9, 10] in machine learning to select differentially expressed genes. Recursive feature elimination method [11–13] is a typical algorithm used in this area. Some classic machine learning algorithms such as decision tree [14], random forest[15] and regression model are able to be used for multiple round training because of their grading feature mechanism. The feature selection algorithm based on penalty term[16] has a regularization term in the model. So the generalization ability of the model is improved significantly. In addition, it can prevent over-fitting. However, the feature selection algorithms which apply to all kinds of data are not applicable to a particular type of data. Therefore, in 2015, Bo Liao et al. proposed a regularization feature ranking method (*l*_2,1_ feature ranking method) [17] for gene expression dataset, which is oriented to label sample data. The algorithm combines multi-objective regression and graphical embedding. By ranking the coefficient matrix, the top ranked genes are selected as differentially expressed genes. Top ranked genes can effectively distinguish disease samples from normal samples. However, many sample labels contain a portion of the weak labels. If the method is used directly to select differentially expressed genes, the performance is unsatisfactory.

According to the accuracy of the label information, the label information of the samples usually includes weak and strong labels. Differentially expressed genes selected from normal samples and disease samples with strong label are more reliable than those selected from normal samples and disease samples with weak label. However, disease samples with weak label also contain some helpful information.

To make optimal use of the label information, we designed a disease-related gene mining method based on weakly supervised learning model. In the first part, to more effectively select differentially expressed genes at different stages of the disease progression, we propose a differentially expressed gene screening method. First, the normal samples are selected as the initial training set with the mid-term-stage and the late-stage disease samples respectively. We use the *l*_2,1_ feature ranking method in [17] to select differentially expressed genes. Then the classifier is used to classify the candidate weak label samples and the samples which are classified to the disease class are iteratively used to extend the differentially expressed gene set until it converges. Therefore, we obtain a differentially expressed gene set at different stages of the disease development. At the same time, it is possible to generalize the feature gene selection ability of the model. In the second part, we propose a difference kernel function utilizing the biological meaning of gene expression data for the differentially expressed gene set. The difference kernel function helps to more accurately represent the relation between the two genes by transforming the expression value features of the initial data under different experimental conditions into the difference features of the expression value changes. We use transductive support vector machine (TSVM) [18] based on the difference kernel function to select disease-related genes in the differentially expressed gene set.

Using Huntington’s disease (HD) gene expression dataset, we demonstrate the effectiveness of the disease-related gene mining method based on the weakly supervised learning model. The weakly supervised learning model selects differentially expressed genes. Gradient boosting decision tree (GBDT) [19] classify the samples using the selected genes. It is demonstrated from the experiments that the weakly supervised learning model has an advantage over other algorithms. The performance of the difference kernel function in distinguishing disease-related genes from non-disease-related genes is good by many different criterion.

## Methods

### The framework of the disease-related gene mining method based on weakly supervised learning model

**Fig. 1:**
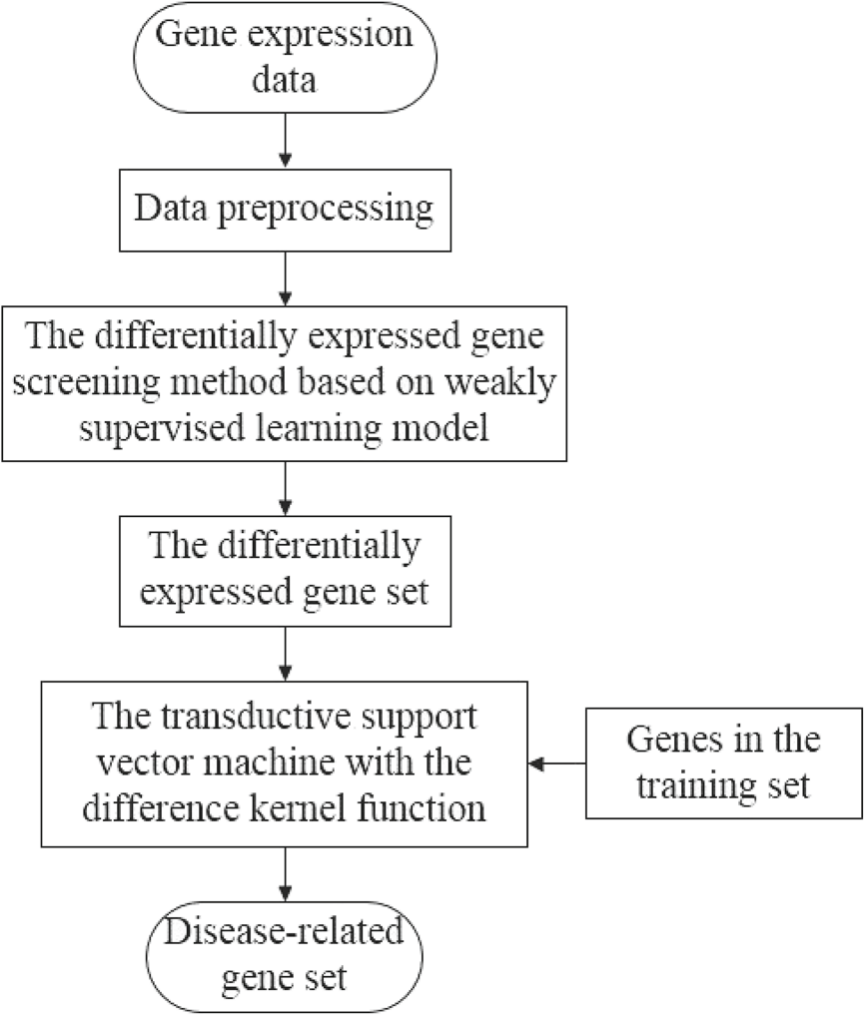
The framework of the disease-related gene mining method based on weakly supervised learning model

In order to screen disease-related genes from the HD gene expression dataset accurately, we designed the following framework of the disease-related gene mining method based on weakly supervised learning model as shown in Fig. 1. The framework consists of the following steps. Firstly, we screen the differentially expressed genes based on the weakly supervised learning model. Then, we screen the disease-related genes in the obtained differentially expressed gene set using TSVM based on the difference kernel function. In the step, the known disease-related gene information is utilized effectively. The difference kernel function we designed can reflect the feature of data objectively and improve the prediction precision.

### The weakly supervised learning model

#### Definition of weak labels

The HD gene expression dataset used in the present study [20] contains six sample labels based on the number of CAG repeats, namely Q20, Q80, Q92, Q111, Q140 and Q175. Samples with Q less than 26 are normal. Samples with Q less than 35 are not sick but the offspring may be sick. There are still some normal samples even though the disease of samples with Q not less than 36 becomes more serious as Q increases. Therefore, the sample label information cannot identify normal samples and disease samples precisely. We assume that samples with Q less than 36 are normal and samples with Q greater than 140 are late-stage disease samples.

In order to extract differentially expressed genes in different stages of disease development, we define the samples of Q175 and Q111 as late-stage and mid-term-stage samples respectively. The samples of Q140, Q92 and Q80 are defined as weak label samples of the late-stage and mid-term-stage disease samples. We defined the above label definition method as a two-label scheme. Although weak label disease samples increase the difficulty of screening differentially expressed genes, it is worth noting that these samples also provide some useful information. In the present study, all the label information is used to screen disease-related genes highly correlated with Q. Therefore, the selected genes may be related to the onset and progression of Huntington’s disease.

#### *l*_2,1_ regularization feature ranking method

*l*_2,1_ feature ranking method is a supervised feature selection method proposed in [17]. We select the top ranked features by sorting the coefficient matrix. The objective function of the *l*_2,1_ feature ranking method as follows [17]:

1 $$ {\begin{aligned} &\min_{W,b}\sum\limits_{i=1}^{m}\parallel W^{T}x_{i}+b-y_{i}\parallel^{2}\\&+\frac{\lambda}{2}\sum\limits_{i,j=1}^{m}\left\| W^{T}x_{i}-W^{T}x_{j}\right\|^{2}S_{i,j}+\gamma\parallel W \parallel_{2,1} \end{aligned}}  $$

Assume that we have m samples and n genes. The gene expression data matrix is *X*=[*x*_1_,*x*_2_···,*x*_*m*_]∈*R*^*n*×*m*^. *x*_*i*_∈*R*^*n*^ means the *ith* sample. *Y*∈*R*^*c*×*m*^ denotes the target matrix. If the *ith* sample belongs to the *jth* class *y*_*j*,*i*_=1, 0 otherwise. For coefficient matrix *W*∈*R*^*n*×*c*^,*w*^*i*^ and *w*_*j*_ denote the *ith* row and the *jth* column of *W*. In the method, heat kernel is used to measure the affinity between samples, as defined in Eq. 2. *ℓ*(*x*_*i*_) represents a set of samples that share the same label with *x*_*j*_.

2 $$ S_{i,j}= \left\{\begin{array}{ll} e^{-\frac{\parallel x_{i}-x_{j}\parallel^{2}}{t^{2}}}& if \ x_{i }\in \ell\left(x_{j}\right) \ or\ x_{j}\in \ell\left(x_{i}\right)\\ 0& otherwise \end{array}\right.  $$

The above objective function [17] consists of three parts. The first part considers the global structure information of the data; The second part considers the local structure information of the data. *l*_2,1_ regularization term is add in the objective function to guarantee that some row coefficients shrink to zero. The coefficients’magnitude can measure the importance of a feature. The top-ranked features are selected by sorting $\left \{{\parallel {w}^{k}\parallel }\right \}_{i=1}^{n}$ in descending order.

#### The differentially expressed genes screening method based on the weakly supervised learning model

**Fig. 2:**
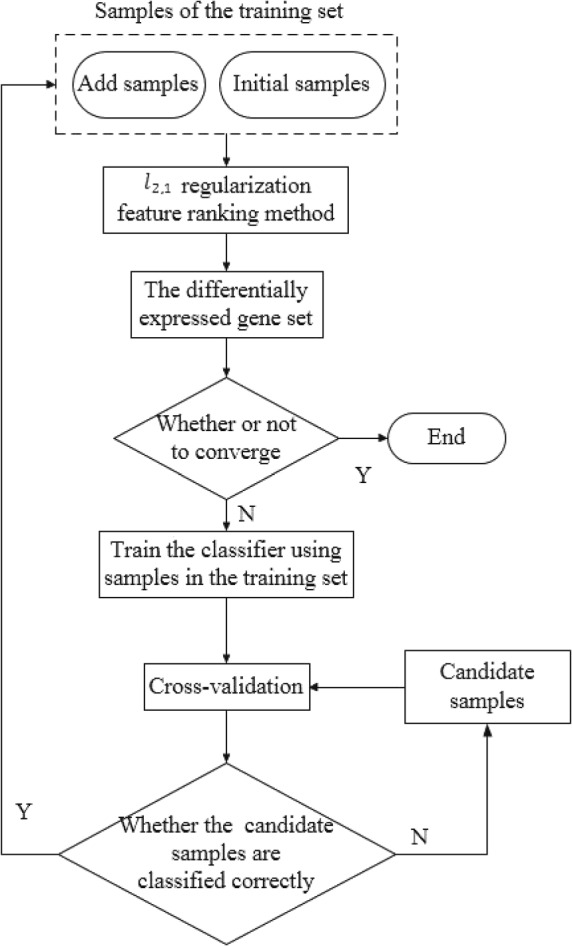
A basic flow chart of the differentially expressed gene screening method based on weakly supervised learning model

The model consists of the following steps. Firstly, we select normal samples and late-stage disease samples (Q175) as the initial training set to screen differentially expressed genes related to late-stage disease. In addition, we select normal samples and mid-term-stage disease samples (Q111) as the initial training set to screen differentially expressed genes related to mid-term-stage disease. Then, we use the *l*_2_,1 feature ranking method to select the feature genes and add them to the differentially expressed gene set. Additionally, the classifier is trained on the training set while the remaining candidate weak label samples are classified. Samples classified to the disease class are added to the training set and repeat the above steps until the differentially expressed gene set converges. Finally, the final set is the intersection of the two differentially expressed gene sets. The above model makes full use of the strong and weak sample label information and generalizes the ability of model to select feature genes, thus a stable and complete differentially expressed gene set is obtained by the model. Figure 2 is a flow chart of the differentially expressed gene screening method based on the weakly supervised learning model.

Based on the above analysis, we summarize the proposed iterative algorithm in Algorithm 1. *M* is a parameter to control the number of differentially expressed genes selected per iteration. *θ* is a parameter to control the convergence condition. It denotes the proportion of the updated differentially expressed genes to the differentially expressed gene set.


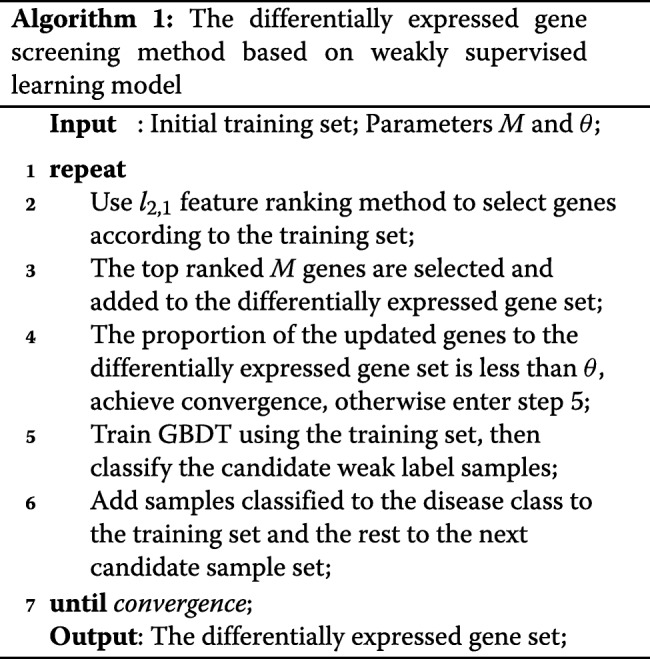


#### The single-label scheme

Weak labels have different selection approaches in our weakly supervised learning model. Defining corresponding weak labels will get more accurate disease-related gene set according the disease progression. In order to verify that screening disease-related genes in the differentially expressed gene set related to disease progression is more accurate, we use Q140, Q111, Q92 and Q80 as the weak label of the late-stage disease samples(Q175) to select differentially expressed genes related to the late-stage disease. We defined the above label definition method as a single-label scheme. Firstly, normal and late-stage disease samples are used as the initial training set. Then, weak label samples classified to the late-stage disease class are added to the training set to select differentially expressed genes through algorithm 1. Although the single-label scheme utilized the supervised effect of the weak label samples in the selection of differentially expressed gene set, it can only obtain a differentially expressed gene set related to the late-stage disease progression. In contrast, the differentially expressed gene set selected by the two-label scheme is more stable and complete because it is related to different stages of the disease progression.

### Transductive support vector machine based on the difference kernel function

Data have domain-specific features. Common kernel functions may have limitations. If the prior knowledge of the domain is known, we can design a mapping function *φ*(*x*) to effectively elevate the result. In HD gene expression dataset, the feature of data is different experimental condition, namely the severity of HD disease according to the number of CAG repeats. Because disease-related genes and non-related genes are linear non-separable in the original gene expression dataset, linear classifiers are not available. We designed a mapping function *φ*(*x*) on basis of understanding the biological knowledge of gene expression data. The mapping function can achieve the transformation from the feature space of the original data to the difference space. In difference space, each feature represents changes between the original adjacent features. In addition, linear classifiers can be used in difference space to achieve better results. The mapping function *φ*(*x*) is formulated as Eq. 3.

3 $$ \varphi(x_{i})=m_{Q_{i}}-m_{Q_{i+1}}  $$

4 $$ m_{Q_{i}}=\frac{1}{n_{Q_{i}}}\sum\limits_{j=1}^{n_{Q_{i}}}{x}_{j}  $$

Where *Q*=[20,80,92,111,140,175]^*T*^ denotes the set of Q. The dimension of the original gene expression dataset is *n*=*n*_20_+*n*_80_+*n*_92_+*n*_111_+*n*_140_+*n*_175_. $ n_{Q_{i}}$ denotes the number of *Q*_*i*_ samples. In Eq. 4, $m_{Q_{i}}$ is the average of samples that share the same Q. We map the original gene expression dataset into a difference space by the mapping function *φ*(*x*). Each new feature represents the difference between the average of two adjacent Q samples, thus the difference kernel function maps the original gene expression dataset to the difference space of expression value changes. In the difference space, the affinity between genes can be measured accurately. With the development of disease, if the changes of expression values are similar, the distance between genes get closer after the functional mapping. It is indicated that the two genes are related during the disease progression. Therefore, differentially expressed genes associated with disease-related genes in the training set are very likely to be disease-related genes. Based on the above biological theory, the classification of genes is more biologically meaningful. Disease-related genes and non-disease-related genes can be better distinguished in the difference space.

We use TSVM [18] based on the difference kernel function (difference_TSVM) to further predict disease-related gene in the differentially expressed gene set. TSVM is a semi-supervised learning method based on support vector machine (SVM) [21]. Thereinto, the choice of kernel function is crucial. In the study, we design a mapping function *φ*(*x*) on basic of the gene expression dataset. The difference kernel function is defined as

5 $$ k\left(x_{i},x_{j}\right)=\varphi(x_{i})^{T}\cdot\varphi\left(x_{i}\right)  $$

In TSVM, we train SVM with the genes in the training set to get parameters. The inner product of the original data in the SVM dual form is replaced by the difference kernel function *k*(*x*_*i*_,*x*_*j*_). Thus, the difference_TSVM method is generated to select disease-related genes.

## Results and discussion

### Gene expression data

The gene expression dataset is about HD, which can be download from http://www.hdinhd.org [20]. The dataset were gotten from the striatum tissue of HD mice by using RNA-seq technology and contains six sample labels based on the number of CAG repeats. There are 48 Q20 samples, 32 Q80 samples, 32 Q92 samples, 32 Q111 samples, 32 Q140 samples and 32 Q175 samples in the dataset. Samples with Q20 are normal. The disease of samples becomes more serious as Q increases for the rest samples. The gene expression dataset is composed of 23,351. Thereinto, some genes have been confirmed whether they are related to disease through biological experiments. The training set contains 88 disease-related genes and 428 non-disease-related genes in the training set.

We conducted a preprocession to filter out noisy and redundant genes. Firstly, We filtered out the genes with expression value of 0, because they are not expressed during the disease progression. Then we select genes with large variance. Finally, we normalize the gene expression data for every sample.

### Evaluation

To verify the performance of the method, we use accuracy, true positive rate (TPR) and false positive (FPR) to evaluate the prediction accuracy of disease-related genes. TPR is defined as the ratio of correctly predicted positive samples to all positive samples. FPR is defined as the ratio of incorrectly predicted samples to all negative samples. Precision is defined as the ratio of correctly positive samples to all the predicted positive samples. Recall is defined as the ratio of correctly positive samples to all the positive samples. The receiver operating characteristic (ROC) curves [22] are created by using TPR and FPR. The precision-recall (PR) curves are created by using Precision and Recall. the area under ROC curves(AUC) and the area under PR curves are used as evaluation criteria for the prediction accuracy of disease-reated genes.

### Performance comparison between differentially expressed gene screening method based on weakly supervised learning model and other algorithms

In the differentially expressed gene screening method based on the weakly supervised learning model, we set the number of differentially expressed genes selected per iteration *M*=1500 and the convergence condition *θ*=0.05 through multiple experiments. For *l*_2,1_ feature ranking method, we set the parameters to default values.

We use the weakly supervised learning model to select differentially expressed genes. Then, GBDT is trained on the differentially expressed gene set to classify samples. For the selection of differentially expressed genes, three competitive methods are chosen for comparison including variance selection method, chi-square test [23] and *l*_2,1_ feature ranking method [17]. In order to ensure fairness, the number of genes selected by the competitive methods is equal to the weakly supervised learning model. We select normal samples and samples at different stages of disease(Q175, Q140, Q111) as the initial training set to select differentially expressed genes respectively. The classifier is trained on the three differentially expressed gene set to classify samples.

**Fig. 3:**
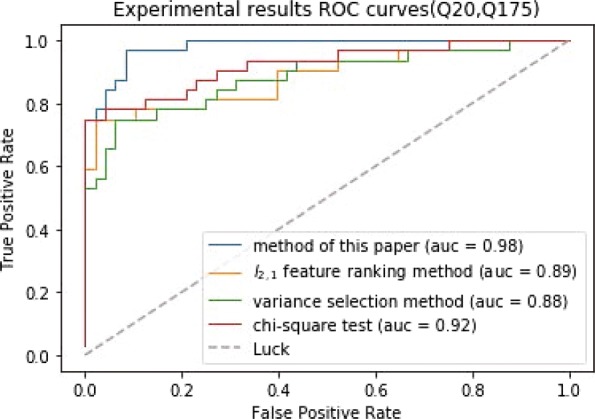
The ROC curves of (Q20, Q175) classification results

**Fig. 4:**
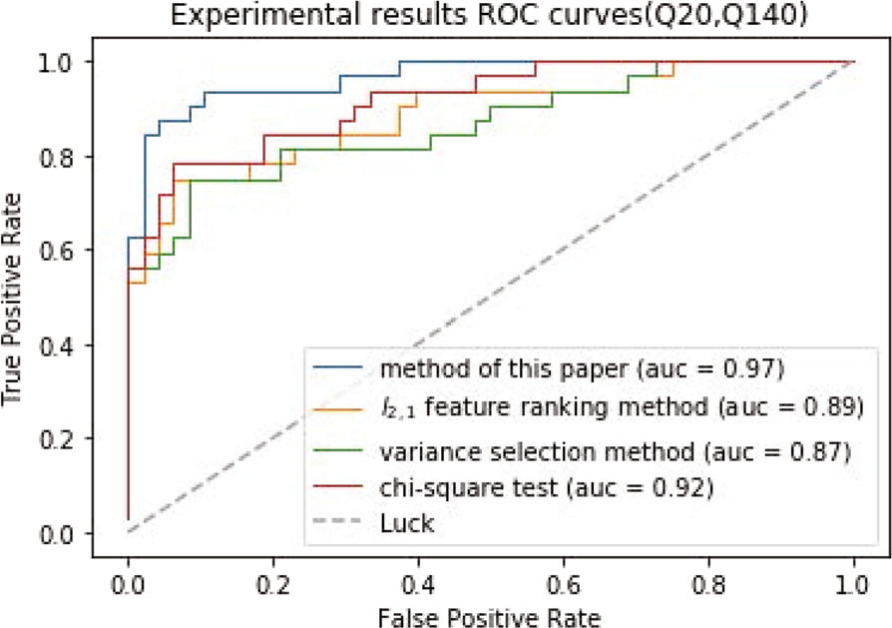
The ROC curves of (Q20, Q140) classification results

**Fig. 5:**
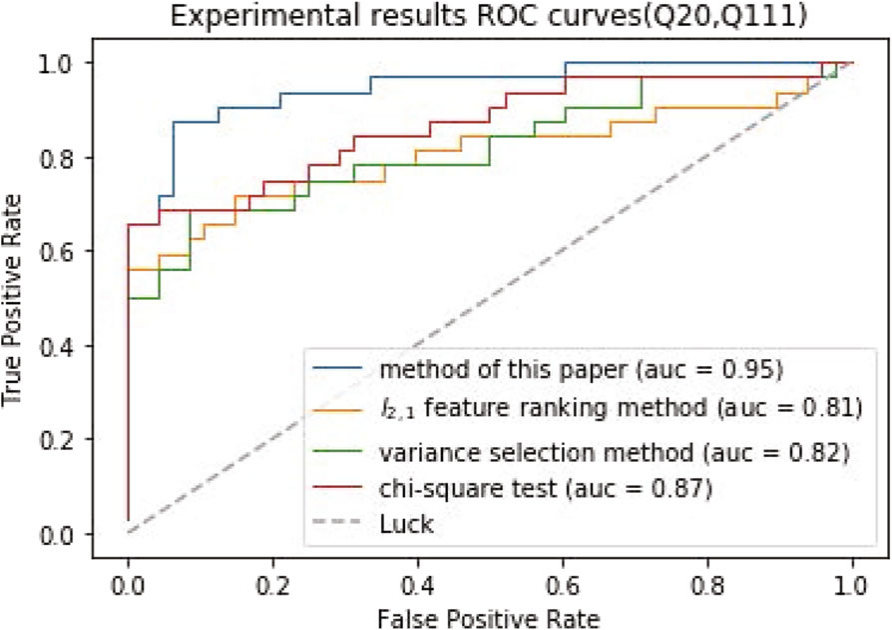
The ROC curves of (Q20, Q111) classification results

The results of experiments are worthless when the Q value of the sample label is small, so we use three groups of disease samples with large Q value to conduct the experiments. In experiments, we use ten fold cross validation to evaluate the classification performance. As can be seen in Figs. 3, 4 and 5, the differentially expressed gene screening method proposed in the study outperforms other methods on the classification performance. Even though chi-square test preforms well, it is necessary to determine the number of differentially expressed genes in advance. However, it is impossible to know that how many differentially expressed genes selected is suitable before experiments. The number of differentially expressed genes selected directly affects the classification results. The experimental results demonstrate that the selected genes are differentially expressed in normal and disease samples and related to the disease progression.

### Convergence analysis of the differentially expressed gene screening method based on the weakly supervised learning model

**Fig. 6:**
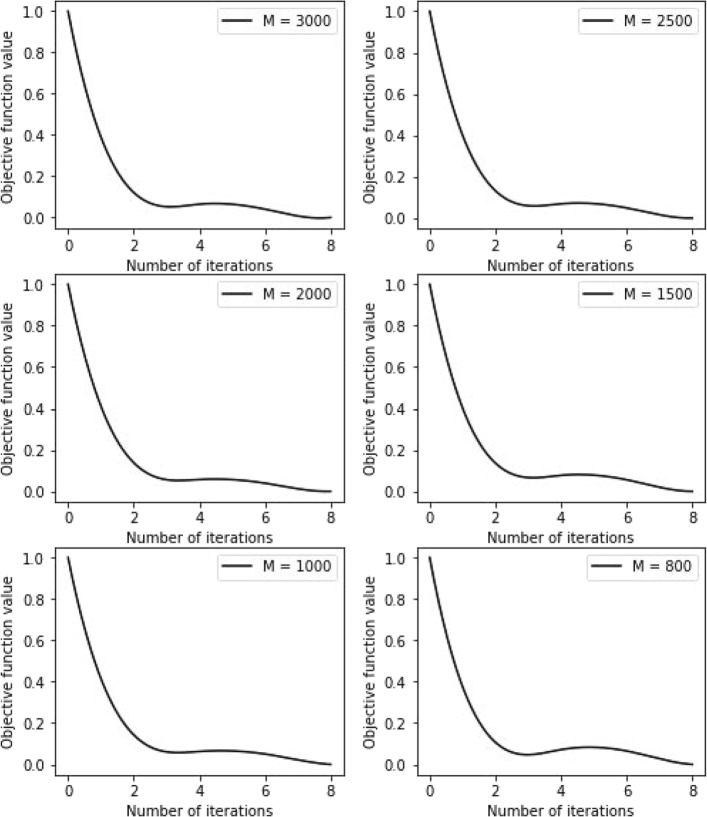
Convergence analysis of differentially expressed gene screening method based on weakly supervised learning model

In Fig. 6, we plot the convergence curves of the model under different parameter *M*. The Y-axis denotes the proportion of the updated differentially expressed genes to the differentially expressed gene set. The X-axis denotes the number of iterations. It can be observed that our model converges quickly and is not sensitive to the value of the parameter *M*.

### Performance comparison between difference kernel function and other kernel functions

In the above differentially expressed gene screening step, 1691 differentially expressed genes were selected according to the two-label scheme and 3136 differentially expressed genes were selected according to the single-label scheme. Then disease-related genes were further selected from the two differentially expressed gene set in the next experiment. The training set is composed of 516 genes, including 428 non-disease-related genes and 88 disease-related genes. We use ten fold cross validation to evaluate the classification performance.

To verify the performance of the difference kernel function in the prediction of disease-related genes, we use the semi-supervised learning model TSVM based on the difference kernel function to select disease-related genes. The method is implemented by the tool of SVM-light [24]. We set the parameters to default values. In addition, we conducted other experiments using the linear kernel function and the radial basis kernel function (rbf).

**Fig. 7:**
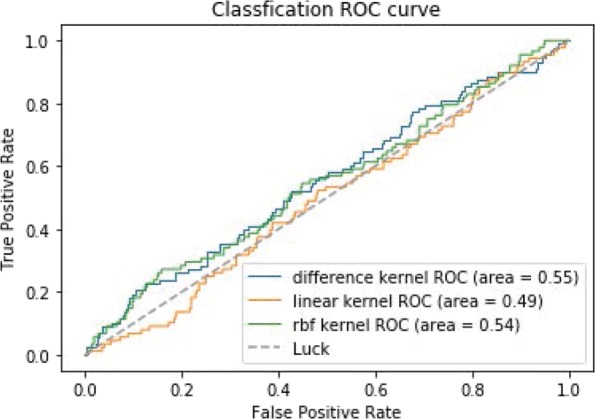
The ROC curves of TSVM classification results based on three kernel functions for the two-label scheme

**Fig. 8:**
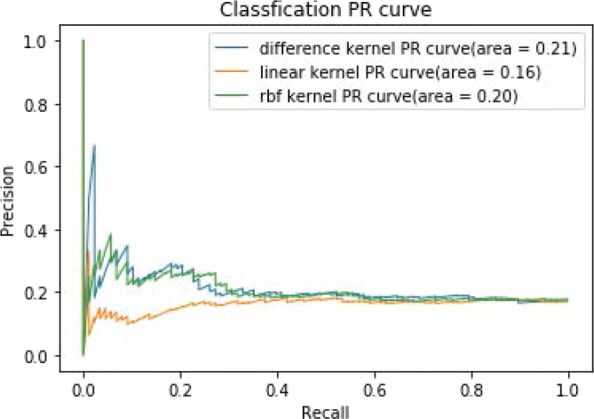
The PR curves of TSVM classification results based on three kernel functions for the two-label scheme

**Fig. 9:**
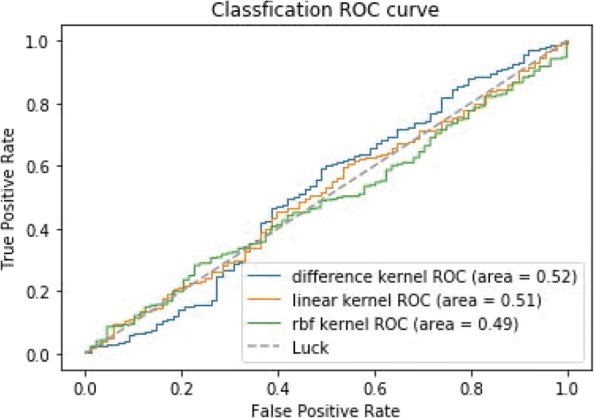
The ROC curves of TSVM classification results based on three kernel functions for the single-label scheme

**Fig. 10:**
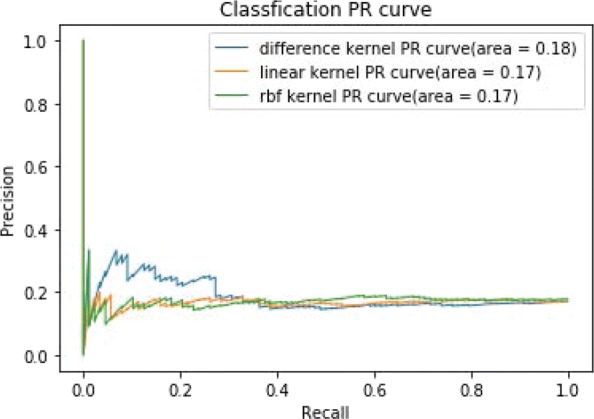
The ROC curves of TSVM classification results based on three kernel functions for the single-label scheme

Figure 7 shows the ROC curves of TSVM based on three different kernel functions for the two-label scheme. However, we can see from Fig. 7 that the AUCs of the three kernel functions are quite low. The closer the AUC is to 1, the better the classification performance. It is indicated that the HD pathological mechanism is complicated, making it challenging to screen disease-related genes only by the traditional algorithm model. Nevertheless, the AUC of the difference kernel function is mildly improved compared with two other kernel functions. Figure 8 shows the PR curves of TSVM based on three different kernel functions for the two-label scheme. The area under PR curve of the difference kernel function is improved compared with two other kernel functions, indicating that the difference kernel function can make disease-related genes rank higher. The prediction precision of the difference kernel function is alse higher for top ranked genes compared with two other kernel functions. As we can see that the PR curves of the linear kernel and rbf kernel fluctuate sharply when the recall rate is small, indicting that they are lack of stability. Figures 9 and 10 shows the classification results of TSVM based on three different kernel functions for the single-label scheme. We can see that the prediction precision of the difference kernel function is higher than two other kernel functions.

From the above experiments, we find that the two-label scheme is superior to the single-label scheme. Because the two-label scheme can obtained a complete set of differentially expressed genes by screening differentially expressed genes in late-stage and mid-term-stage of disease progression. Therefore, a more accurate disease-related gene set can be obtained by difference_TSVM using the two-label scheme.

**Fig. 11:**
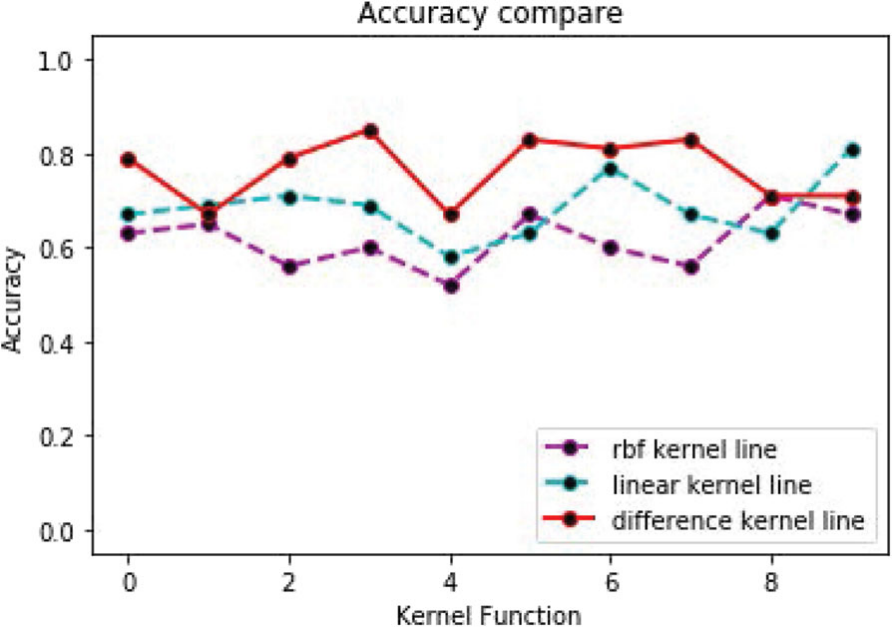
The accuracy of 10-fold cross-validation for the two-label scheme

**Fig. 12:**
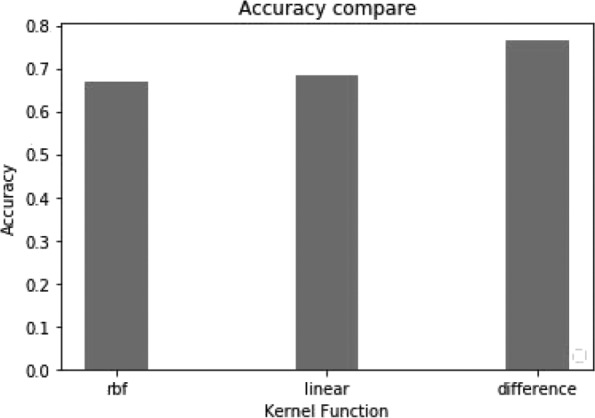
Comparison of average classification accuracy for the two-label scheme

The following experiments analyze the prediction accuracy of the two-label scheme. From Fig. 11, we can see the accuracy of each group in the ten fold cross validation based on different kernel functions. The prediction accuracy of the difference kernel function is relatively stable and high compared with two other kernel functions. It is indicated that difference_TSVM has good robustness. From Fig. 12, we can see the average prediction accuracy based on different kernel functions. The X-axis denotes different kernel functions. The Y-axis denotes average prediction accuracy. The average prediction accuracy of the difference kernel function is 0.766, then linear kernel function is 0.685, then rbf kernel function is the lowest (0.67).

In conclusion, the performance of the difference kernel function is better than other kernel functions in selecting disease-related genes. Because the difference kernel function considers the biological knowledge in gene expression dataset. The two-label scheme is superior to the single-label scheme. Therefore, screening differentially expressed gene set at different stages of the disease development is helpful to obtain an accurate disease-related gene set.

### Performance comparison between transductive support vector machine method based on difference kernel function and other algorithms

**Fig. 13:**
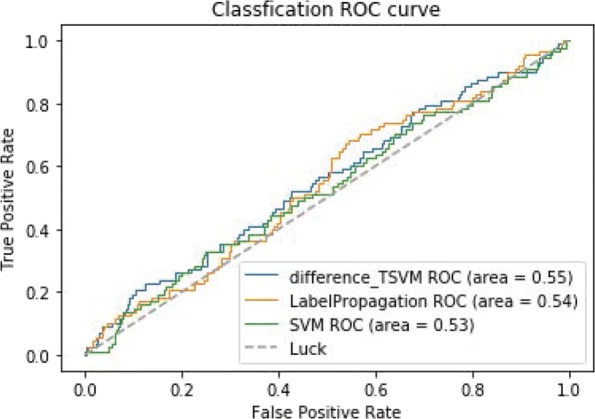
The ROC curves of classification results for the two-label scheme

**Fig. 14:**
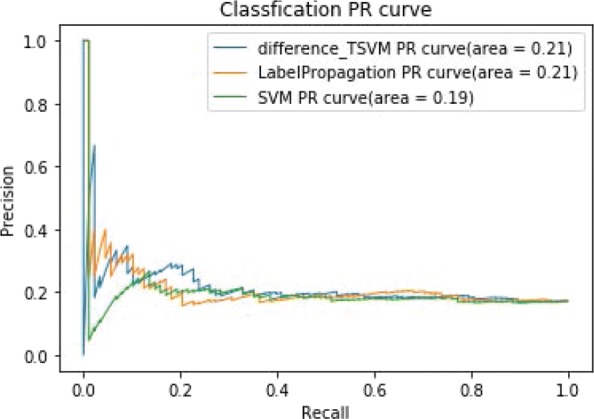
The PR curves of classification results for the two-label scheme

**Fig. 15:**
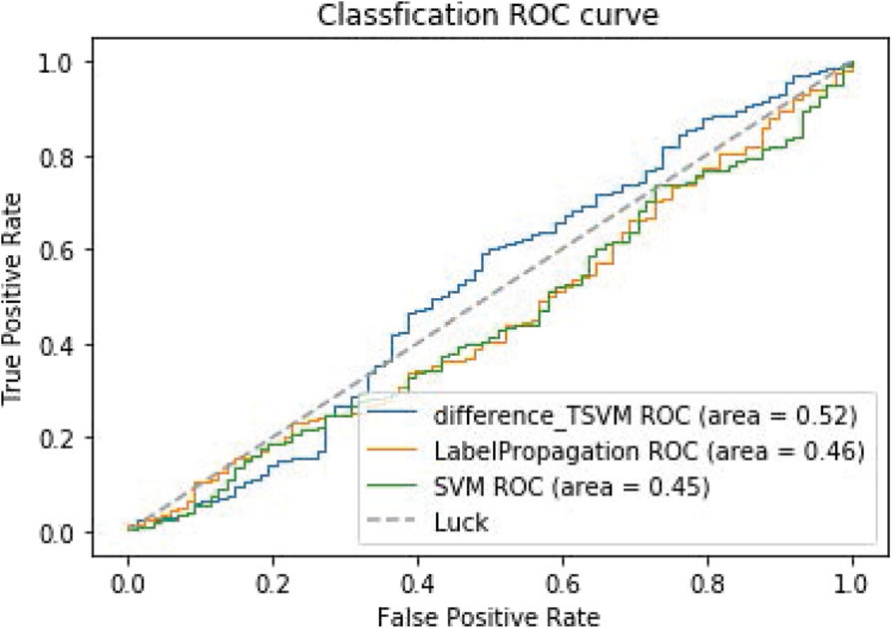
The ROC curves of classification results for the single-label scheme

**Fig. 16:**
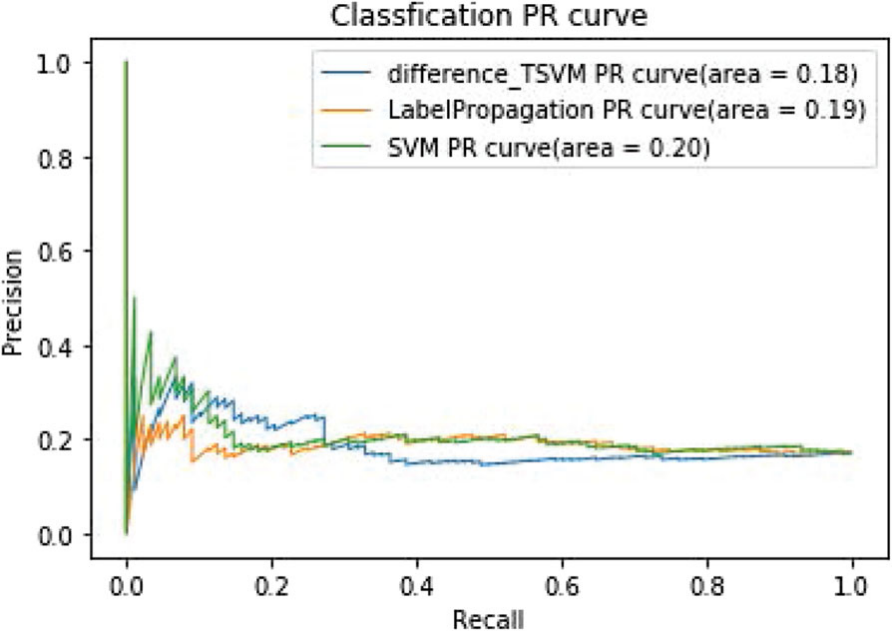
The PR curves of classification results for the single-label scheme

In order to further verify the performance of difference_TSVM, we compare it with SVM based on rbf and label propagation method based on semi-supervised learning model [25]. From Fig. 13, we can see that the ROC curves of the three methods are similar. The prediction accuracy of difference_TSVM is higher than two other methods. Because the difference kernel function can map the original gene expression dataset to the difference space. Linear classifiers can classify disease-related genes and non-disease-related genes accurately. The classification accuracy of the label propagation is higher than SVM based on rbf because the semi-supervised method can make full use of the unlabeled data in the dataset to predict disease-related genes. From Fig. 14, we can see the PR curves of the three method. The classification performance of difference_TSVM is improved compared with two other methods when the recall rate is less than 0.3, indicating that the prediction accuracy for top ranked genes of difference_TSVM is higher. From Figs. 15 and 16, we can see the ROC and PR curves of the experimental results for the single-label scheme. We can know that the performance of difference_TSVM is better than two other methods.

From the above experiments, the difference_TSVM has advantages in the prediction disease-related genes. Compare two groups of experiments, the classification results for the two-label scheme are more accurate. It is indicated that considering weak and strong label information of samples to extract differentially expressed genes at different stages of disease development can obtain a more accurate disease-related gene set.

The experiments demonstrate that difference_TSVM outperform other methods in the prediction of disease-related genes through the comparison and analysis of the experimental results using the above two evaluation criteria. The known label genes and differentially expressed gene set were used as the training set. We used difference_TSVM to select disease-related genes in the differentially expressed gene set. Finally, we select 363 disease-related genes to further analyze molecular of disease.

## Conclusion

In the study, we designed a disease-related gene mining method based on the weakly supervised learning model. We separated the model into two steps. In the first step, the differentially expressed genes are screened based on the weakly supervised learning model. In the model, the strong and weak label information at different stages of the disease development is fully utilized. The final differentially expressed gene set is the intersection of the two differentially expressed gene sets. In our model, differentially expressed genes related to the disease development is selected accurately. We can distinguish disease samples from normal samples using the selected genes. In the second step, we proposed the difference kernel function to map the original data to the difference space. In the difference space, the affinity between genes can be measure more accurately. Then, we use TSVM based on the difference kernel function to classify differentially expressed genes. The experiments firstly demonstrate that the differentially expressed genes screening method is effective. It also suggests that the performance of two-label scheme is better than single-label scheme. The difference kernel function achieves better performance compared with other kernel functions. Finally, the analysis of the expermental comparison verifies the disease-related gene mining method based on the weakly supervised learning model improved the prediction accuracy compared with other methods.

## Data Availability

The gene expression data used in this study were downloaded from http://www.hdinhd.org.

## Abbreviations

AUC: The area under the ROC curve
CAG: Cytosine adenine and guanine
difference_TSVM: Transductive support vector machine with difference kernel function
FDR: False discovery rate
FPR: False positive rate
GBDT: Gradient boosting decision tree
HD: Huntington’s disease
*l*_2,1_ feature ranking method: *l*_2,1_ regularization feature ranking method
P: Precision
PR: Precision-recall
R: Recall
ROC: Receiver operating characteristic
SAM: Significance analysis of microarrays
SVM: Support vector machine
TPR: True positive rate
TSVM: Transductive support vector machine
